# A Study on the Validity of a Computer-Based Game to Assess Cognitive Processes, Reward Mechanisms, and Time Perception in Children Aged 4-8 Years

**DOI:** 10.2196/games.5997

**Published:** 2016-09-22

**Authors:** Janneke CAW Peijnenborgh, Petra PM Hurks, Albert P Aldenkamp, Erik D van der Spek, GWM Rauterberg, Johan SH Vles, Jos GM Hendriksen

**Affiliations:** ^1^KempenhaegheCenter for Neurological Learning DisabilitiesHeezeNetherlands; ^2^Maastricht UniversityFaculty of Psychology and NeuroscienceMaastrichtNetherlands; ^3^KempenhaegheDepartment of Research and DevelopmentHeezeNetherlands; ^4^Eindhoven University of TechnologyFaculty of Electrical EngineeringEindhovenNetherlands; ^5^Eindhoven University of TechnologyFaculty of Industrial DesignEindhovenNetherlands; ^6^Maastricht University Medical CenterDepartment of NeurologyMaastrichtNetherlands

**Keywords:** experimental games, ADHD, children, neuropsychological test

## Abstract

**Background:**

A computer-based game, named Timo’s Adventure, was developed to assess specific cognitive functions (eg, attention, planning, and working memory), time perception, and reward mechanisms in young school-aged children. The game consists of 6 mini-games embedded in a story line and includes fantasy elements to enhance motivation.

**Objective:**

The aim of this study was to investigate the validity of Timo’s Adventure in normally developing children and in children with attention-deficit/hyperactivity disorder (ADHD).

**Methods:**

A total of 96 normally developing children aged 4-8 years and 40 children with ADHD were assessed using the game. Clinical validity was investigated by examining the effects of age on performances within the normally developing children, as well as performance differences between the healthy controls and the ADHD group.

**Results:**

Our analyses in the normally developing children showed developmental effects; that is, older children made fewer inhibition mistakes (*r*=−.33, *P*=.001), had faster (and therefore better) reaction times (*r*=−.49, *P*<.001), and were able to produce time intervals more accurately than younger children (ρ=.35, *P*<.001). Discriminant analysis showed that Timo’s Adventure was accurate in most classifications whether a child belonged to the ADHD group or the normally developing group: 78% (76/97) of the children were correctly classified as having ADHD or as being in the normally developing group. The classification results showed that 72% (41/57) children in the control group were correctly classified, and 88% (35/40) of the children in the ADHD group were correctly classified as having ADHD. Sensitivity (0.89) and specificity (0.69) of Timo’s Adventure were satisfying.

**Conclusions:**

Computer-based games seem to be a valid tool to assess specific strengths and weaknesses in young children with ADHD.

## Introduction

Assessment of children’s cognitive strengths and weaknesses is an important focus of clinical child neuropsychological research and clinical care worldwide [1]. Cognitive abilities are quantified traditionally by use of, for example, paper-and-pencil performance tests and more recently with computer-assisted tools [2,3]. Performances on traditional cognitive tests are believed to be influenced significantly by noncognitive functions, such as motivation and perseverance [4]. Therefore, a lower score on, for instance, a working memory test might indicate a memory problem but also, for example, a decreased motivation. This overall performance score therefore only limitedly reflects the underlying “cause” in case of a decreased performance, which makes this score difficult to interpret. In order to test one’s cognitive abilities more purely, it would be preferable to (1) optimize motivation in the test situation and (2) assess motivation in a separate test additionally— unfortunately, tests measuring motivation in children are relatively scarcely used in the clinics.

On the basis of the literature, it is known that introducing immediate (vs delayed) rewards and the adaptation of item difficulty levels to the child’s abilities are likely to increase motivation in children and help the child to stay focused on the tasks that he or she needs to do [5]. Also, introducing a context (eg, by introducing cognitive tests in the context of a story) may increase motivation, although data on the effects of including a story line on motivation or engagement in games are so far inconclusive [6]. The introduction of a story line improves the child’s feeling of being part of a gaming environment [7]. In addition, the use of intrinsic fantasy elements has been found to improve motivation to conduct a specific task [8,9].

The above-mentioned increase in popularity of computer-assisted assessment tools is partly caused by the fact that it is relatively easy to implement these immediate rewards, to adapt difficulty levels, to implement a story, and to use intrinsic fantasy elements. This leads to a situation in which the child does not have the feeling of being assessed but instead thinks that he or she is playing a game [10]. This is especially important in tests that are designed for children because it is known that children can behave differently when they know they are being studied (also known as the Hawthorne effect [11]). For this purpose, we developed “Timo’s Adventure,” a computer-based game that consists of 6 mini-games. These mini-games aim to assess different cognitive processes, for example, attention, planning, and working memory; delay aversion, as a measure of motivation; and time perception (see method section). The aim of this study was to examine the validity of Timo’s Adventure in assessing strengths and weaknesses in the above-mentioned domains of young children, aged 4-8 years. Proof of validity was sought by studying group differences in performance on the mini-games. Two types of relevant group comparisons were made. First, the age of the child is believed to be a relevant variable and was therefore studied in relation to performances on the mini-games: we expected younger children (aged <6 years) to perform less well than older children on all mini-games (in line with studies on, eg, cognitive development [12] and time perception [13,14]). For this purpose, age-related differences in a group of normally developing children (N=96) were examined.

Next, the scores on the mini-games of normally developing children were compared with those of children with attention-deficit/hyperactivity disorder (ADHD; N=40). Attention-deficit/hyperactivity disorder is a developmental disorder that is associated with academic difficulties and social disadvantage [15]. According to the *Diagnostic and Statistical Manual of Mental Disorders* (Fifth Edition; *DSM-V*) [16], 2 main areas of impairment in children with ADHD exist: inattention (eg, difficulty in maintaining attention during a task or problems in dividing attention) and hyperactivity and impulsive behavior (eg, acting out before thinking about the consequences). Previous research has found that cognitive difficulties, more specifically in the domain of working memory and attention, occur in children with ADHD [17]. However, according to the triple-pathway model by Sonuga-Barke et al [18], not all children with ADHD have cognitive weaknesses. In this model 3 distinct patterns of ADHD deficits are distinguished. The first pathway is related to cognitive functions and is called the inhibitory-based executive dysfunction. This pathway views ADHD as a disorder of dysregulation of thought and action associated with diminished inhibitory control (ie, executive functions). In the second pathway, ADHD is explained as a motivational style associated with fundamental alterations in reward mechanisms. Children with ADHD are assumed to prefer small immediate rewards over large delayed rewards, which results in inattentive, overactive, and impulsive behaviors [19]. The third pathway states that deficits in time perception, for instance, deficits in distinguishing between two time intervals, producing time intervals, and estimating time, are another component of ADHD. Indeed, time perception deficits have been reported for children with ADHD [20]. However, the results are not consistent; that is, some authors report no ADHD-related deficits [21]. All 3 pathways are believed to have their own neural substrate [18]. Sonuga-Barke and colleagues [18] found in children with ADHD aged between 6 and 17 years that delay aversion, poor executive functions, and poor time perception are core, but unrelated and independent, characteristics of ADHD. A person with ADHD can have deficits in one of the pathways or a combination of pathways. Neuropsychological measurements (which are used to examine possible deficits in one of the pathways) usually focus primarily on just one of the pathways, whereas one can conclude from the model by Sonuga-Barke et al that it is necessary to gain information on possible deficits in all 3 pathways. Sonuga-Barke and colleagues used several distinctive computerized tasks to collect information about the 3 pathways. These were, however, not connected in a fantasy gaming environment or by a story line. In our game, a story line was included in order to immerse the player in an intrinsic fantasy and possibly improve the reliability of the diagnosis. To our knowledge no computerized diagnostic tools exist in which all 3 pathways are included in combination with all motivation-enhancing elements discussed above (including a story line or fantasy game elements), although some training tools with a story line exist (for instance, Braingame Brian [22]). In Timo’s Adventure story line, distracting factors are included to measure real-life distraction. Previous research found that distractors in a computerized continuous performance test resulted in more distractibility in children with ADHD than in their healthy peers [23].

In summary, the aim of this study was to investigate the clinical validity of Timo’s Adventure.

## Methods

### Participants

#### Normally Developing Children

Parents of all children enrolled in the first 4 grades of 4 Dutch elementary schools were informed by a letter about the study. Informed consent of 102 children was acquired. A total of 4 children were excluded because they were not native speakers and instructions in Timo’s Adventure were in the Dutch language. In addition, 2 children were excluded because they had a *DSM-V* diagnosis. The final dataset consisted of 96 children (43 boys), age ranging from 4 to 8 years. An overview of characteristics for this group can be found in Table 1.

All children were tested individually in a private room at their school. Approval for testing this sample was given by the Ethical Review Committee of the Faculty of Psychology and Neuroscience of Maastricht University, the Netherlands.

#### Children With Attention-Deficit/Hyperactivity Disorder

Parents of patients of the outpatient clinic Center for Neurological Learning Disabilities were asked to participate in this study by their medical specialist. In parallel, parents of children enrolled in a special needs program for children with behavioral problems were informed by a letter about the study and asked to participate, via the children’s school. Informed consent of 62 children with a diagnosis of ADHD was acquired. A total of 22 children did not meet the inclusion criteria because they had a comorbid *DSM-V* diagnosis (n=4), because they used medication for attentional problems and hyperactive behavior (stimulants, atomoxetine, tricyclic antidepressants, or clonidine; n=6), or because of a combination of these exclusion criteria (n=12). The final dataset of the ADHD group consisted of 40 children (30 boys), all with a diagnosis of ADHD according to *DSM-V*. These diagnoses were made on the basis of a protocol formulated by Goldman et al [24] that includes (1) extensive history taking, (2) cognitive testing, (3) general physical and neurological examination of the child, and (4) systematic assessment of ADHD characteristics by means of structured questions based on the most recent version of the *DSM* [16]. Age range of the clinical sample was 6-8 years. An overview of characteristics of the ADHD group can be found in Table 1.

Children enrolled in the special needs program for children with behavioral problems were tested individually in a private room at their school. The children who were patients of the Center for Neurological Learning Disabilities were seen for neuropsychological testing as part of clinical care.

Approval for testing this sample was given by the Medical Ethical Board of Kempenhaeghe.

**Table 1:** Characteristics of all participants.

Characteristics	Typically developing children	ADHD^a^ group
Number of participants	96	40
Boys/girls	43/53	30/10
Age range, years	4-8	6-8
Mean age (SD), years	5.85 (1.33)	6.90 (0.74)
Verbal IQ (WPPSI-III-NL^b^ Vocabulary), mean (SD)	94.74 (10.79)	87.92 (13.05)

^a^ADHD: attention-deficit/hyperactivity disorder.

^b^WPPSI-III-NL: Dutch version of the Wechsler Preschool and Primary Scale of Intelligence [25].

### Materials

#### Computer-Based Game Timo’s Adventure

Timo’s Adventure is a single-player game. All tasks are embedded in a story line: the main character in Timo’s Adventure is Timo, a friendly alien whose rocket has run out of fuel [26]. He asks the child to go on an adventure together to collect stars that can be used as fuel. To complete all tasks, it takes approximately 20 minutes. The game is categorized as a serious game: a game designed for specific purposes beyond entertainment [27]. The game has a first-person view to simulate the feeling of presence, to make the child feel like he or she is inside the game world.

Development of the game was divided into 3 stages: design, implementation, and evaluation, as based on the iterative software cyclic model and the spiral model [28-30]. Besides the designers of Eindhoven University of Technology, users were invited in the design process. Users in this case were children (who helped us by explaining what they would like and by making drawings) and psychologists from Kempenhaeghe (who participated in the development of the functionalities of the game, the visual graphics, and story line that needed to match the age of the children). In the implementation phase, the functionalities of the game were created (by engineers of Eindhoven University of Technology in close collaboration with the psychologists from Kempenhaeghe). During the evaluation phase (consisting of a user test with a paper prototype technique and an computer-based prototype), the game was played and evaluated by children and by psychologists who were not part of the development team. This feedback was used to improve the game and remove small bugs [31].

The computer-based game consists of 6 different tasks (ie, 6 mini-games), each of which measures different neurocognitive functions and thus gives information about possible deficits. The 6 tasks measure aspects of executive functions, time perception, and reward mechanisms to represent the 3 pathways of Sonuga-Barke’s model. All tasks were developed because of the need to measure the specific function and modeled after the theoretical background of the triple-pathway model.

##### Pathway 1: Executive Functions

All irrelevant mouse clicks are measured and thus give information on impulsivity and hyperactive behavior of the child. Furthermore, the following mini-games are included to assess impairments in this pathway: Dressing up (planning), Sandwich (working memory), Monkey (inhibition), and Magic Land (simple reaction time).

**Figure 1:**
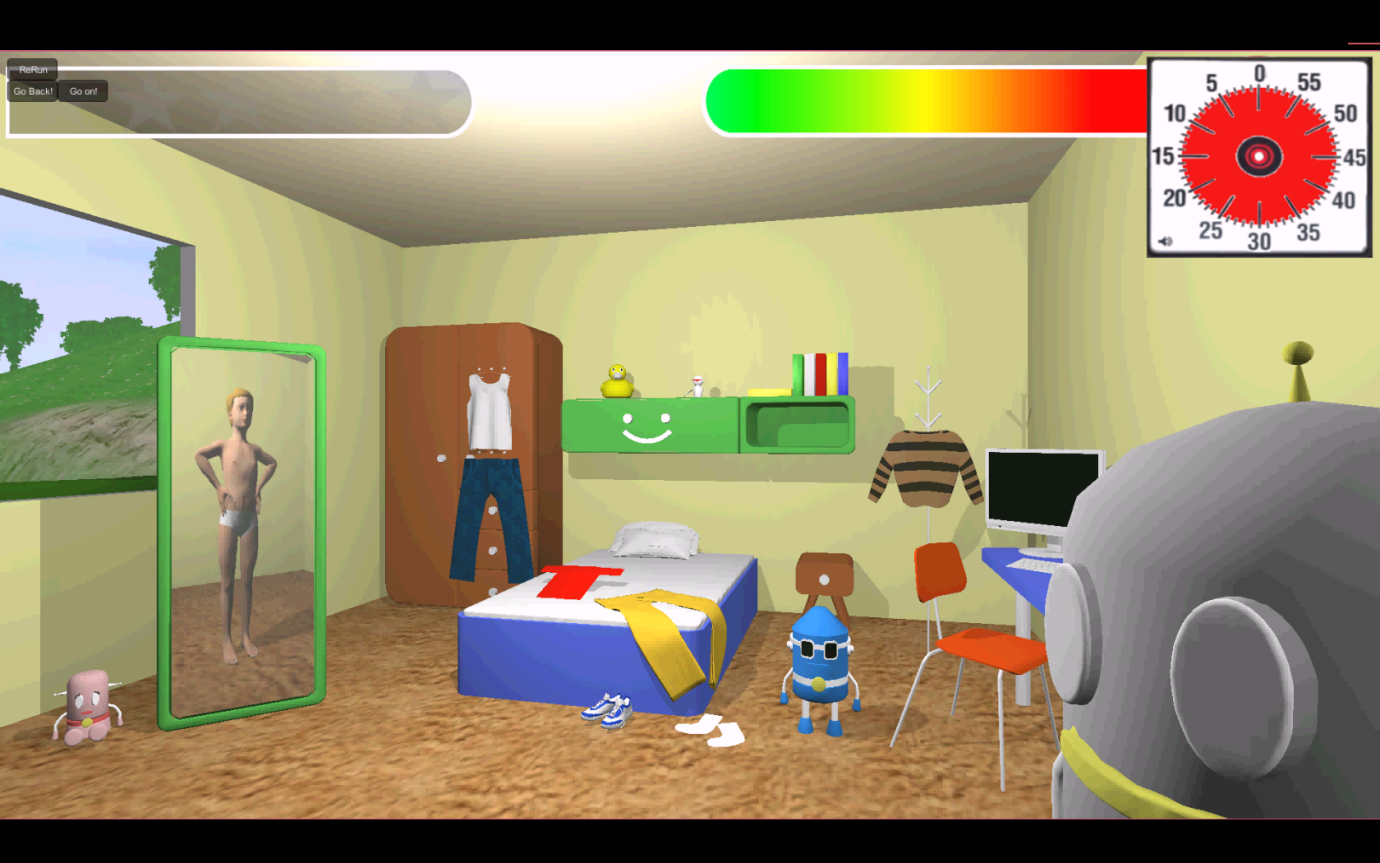
Screenshot of the mini-game Dressing up (planning).

The first task, the mini-game Dressing up (planning), is set in the bedroom of the child. Timo tells the child that he or she needs to get dressed before the adventure can start. Several garments are spread throughout the room, which can be selected by clicking on them (see Figure 1). After clicking, the garment moves toward the reflection of the child in the mirror and the child gets dressed. This task gives information on the planning and organization skills of the child. The order in which the child selects the clothes is assessed to see whether the child is capable of planning his or her actions in the right order. The child can get a total score of 2 points: 1 point for being completely dressed and 1 point for using an executable and correct manner.

**Figure 2:**
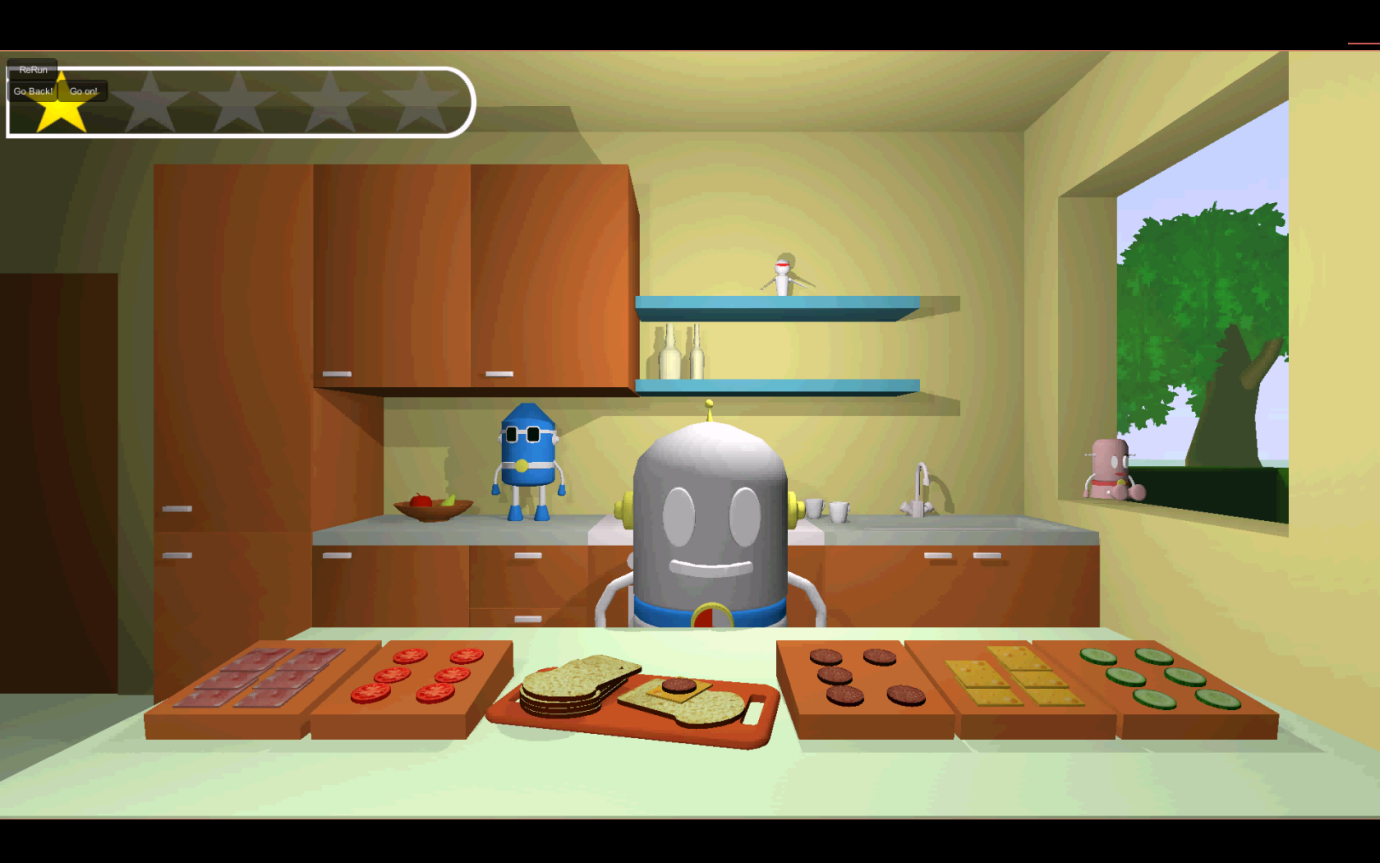
Screenshot of the mini-game Sandwich (working memory).

The second task, the mini-game Sandwich (working memory), is set in the kitchen (see Figure 2). Timo tells the child that it is necessary to eat something before starting the adventure, and he shows the child pictures of the ingredients. The child needs to remember these ingredients and select them in the same order as presented by Timo. The first sandwich starts with 2 ingredients, adding up to 5 ingredients in the last sandwich. This task is a measurement of the capacity of the visual working memory. According to Craeynest [32], the capacity of the working memory develops from remembering 2 targets when the child is 2.5 years old, to 3 targets when 3 years old, and 5 targets when the child is 7 years old. Martinussen et al [33] found that the capacity of the working memory in children with ADHD is markedly lower. In this task, the child can get a total score of 5 points: one for each correct sandwich.

**Figure 3:**
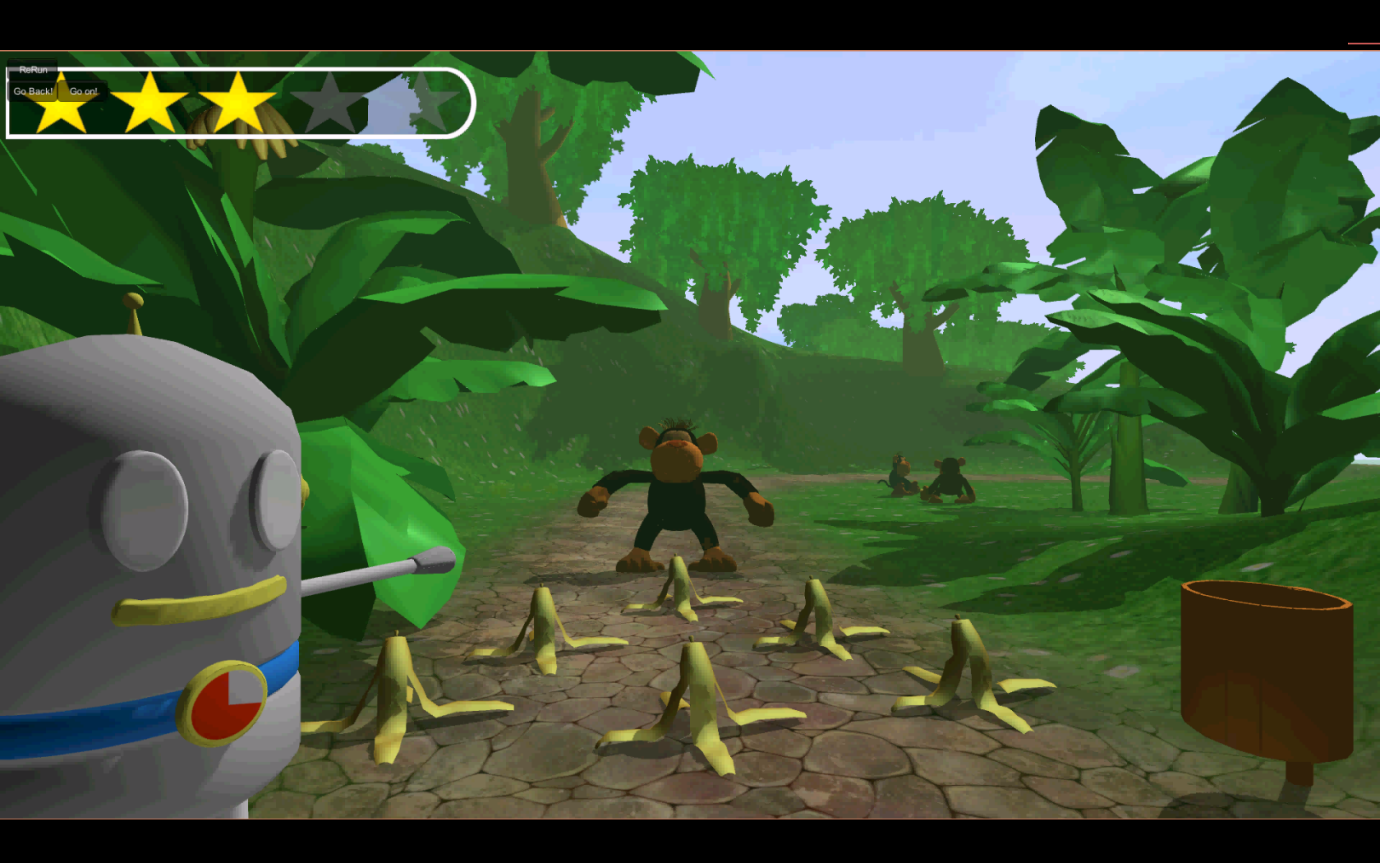
Screenshot of the mini-game Monkey (inhibition).

In the third task, the mini-game Monkey (inhibition), a monkey has thrown banana peels on the road (see Figure 3). To cross the road, the child needs to swipe the banana peels and clear the road. However, if the monkey sees the child swiping banana peels, it will undo the child’s actions. The monkey is playing hide-and-seek and appears suddenly. The child needs to wait for the moment the monkey disappears. This is a go or no-go task that gives information on the response inhibition of the child: is the child capable of inhibiting his or her response until the monkey hides? Children with ADHD have deficits in this response inhibition and they can be inclined to react impulsively [19]. The number of failures (ie, when the monkey sees the action of the child) is the outcome variable of this task; the higher this score, the worse the inhibition skills.

**Figure 4:**
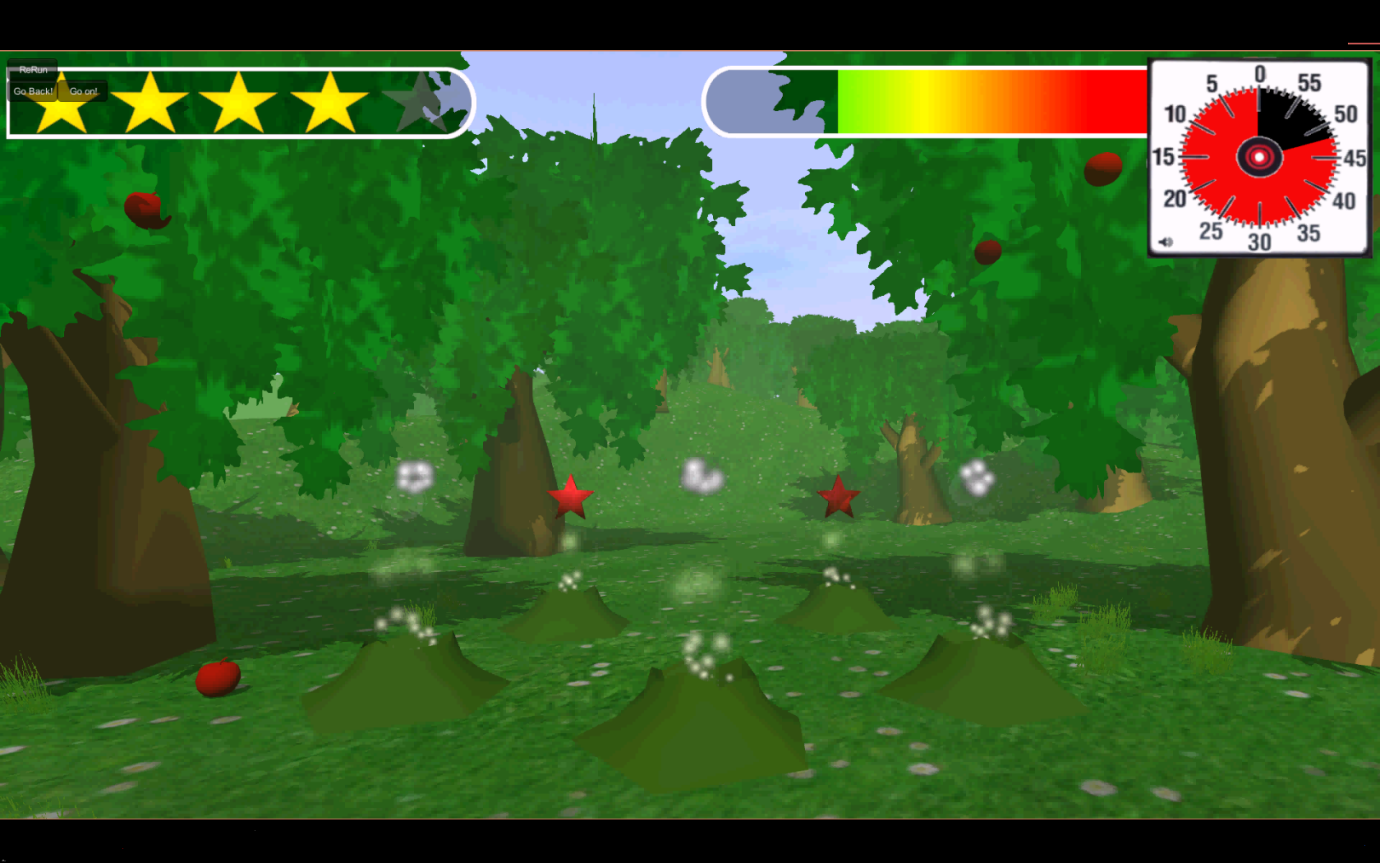
Screenshot of the mini-game Magic Land (simple reaction time).

In the mini-game Magic Land (simple reaction time), stars shoot upward from magic holes (see Figure 4). The child needs to collect these stars. If the child does not react within 2 seconds, the stars will disappear. The task ends after 50 stars shoot upward. Outcome variables in this task are the number of collected stars (with a maximum of 50) and average reaction time for collected stars. Slower and more variable reaction times have been found to be a characteristic of ADHD [34].

**Figure 5:**
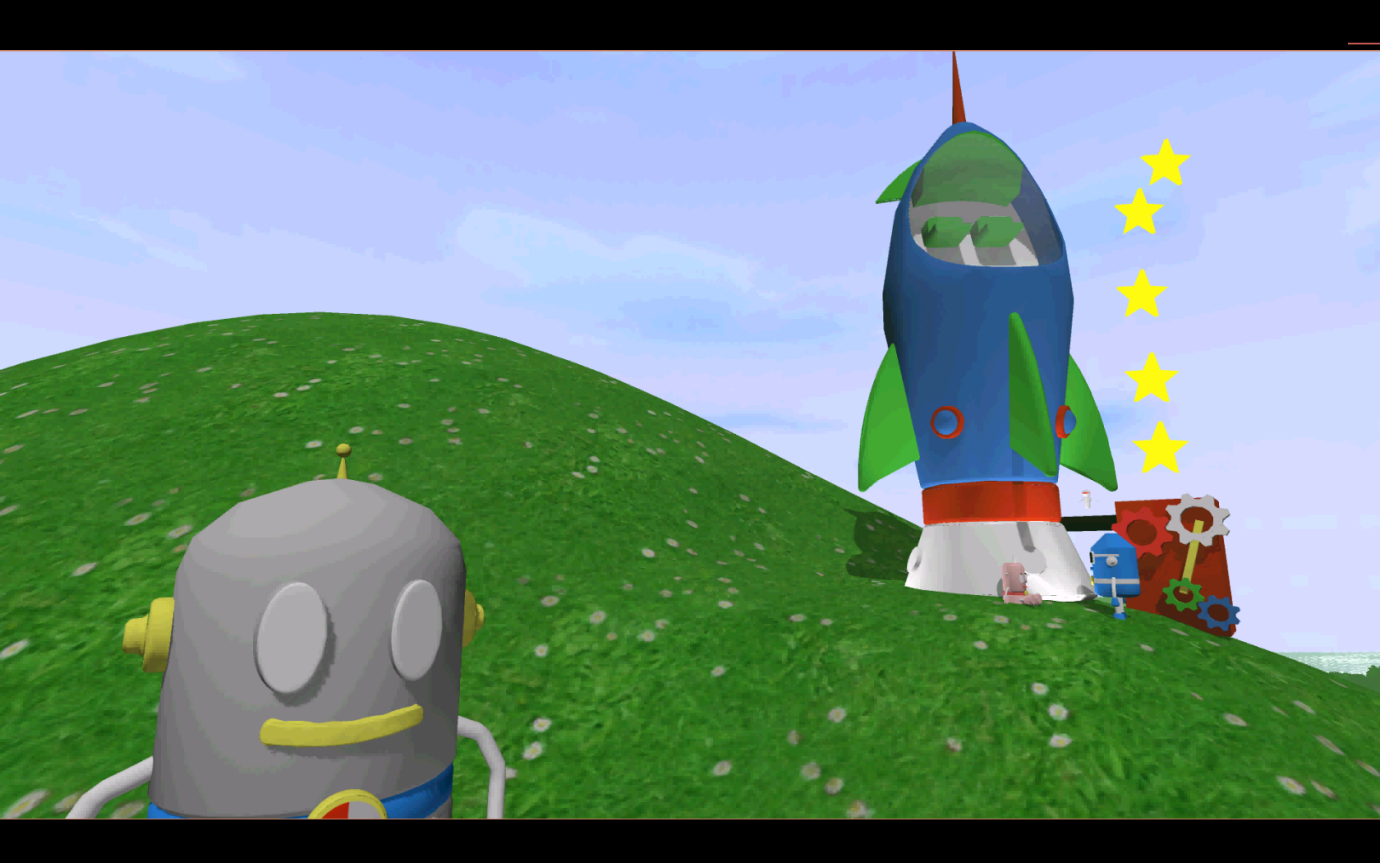
Screenshot of the mini-game Rocket (reward mechanisms).

##### Pathway 2: Reward Mechanisms

In the task mini-game Rocket (delay aversion; see Figure 5), the child gets a choice between an immediate but small reward (ending of the task) or a delayed but bigger reward (a flight in the rocket, after 2 minutes of waiting). The child can end the task at any moment. Impulsive behavior occurs when responding produces more immediate, relatively smaller rewards at the cost of delayed, larger rewards [35]. Outcome variables are whether the child chooses a small or big reward and how long (in seconds) the child waited.

##### Pathway 3: Time Perception

**Figure 6:**
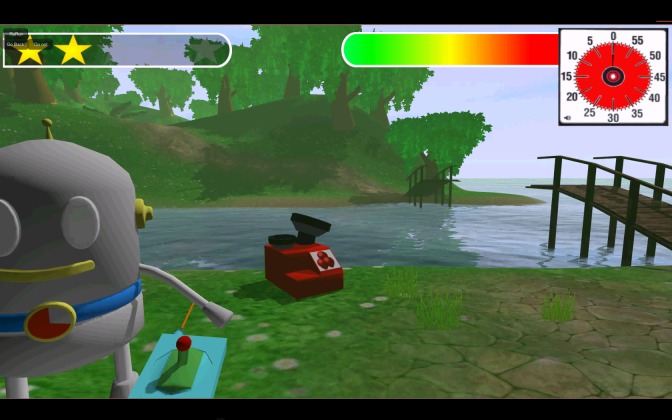
Screenshot of the mini-game Balloon (time perception).

The task mini-game Balloon (time production) is set at a river with a broken bridge (see Figure 6). To cross the river, the child needs to inflate a balloon with the balloon machine by producing a time interval of 10 seconds. A produced interval between 9 and 11 seconds results in a perfect balloon. When the produced interval is smaller than 9 seconds the balloon falls into the water, and a produced interval larger than 11 seconds results in a balloon that flies away. The child can make a maximum of 3 perfect balloons, or the task will end after 3 minutes with a perfect balloon (regardless of what the interval is). Barkley et al [36] suggested that the estimation of temporal intervals is atypical in children with ADHD. The number of correct balloons is an outcome variable. Furthermore, the average production interval for the first 3 balloons is measured by subtracting 10 seconds from each of the first 3 balloons, transforming these scores to absolute scores, adding these scores, and then dividing them by 3. The higher this score, the less precise the mean produced intervals are.

#### Vocabulary

This subscale of the Dutch version of the Wechsler Preschool and Primary Scale of Intelligence (WPPSI-III-NL [25]) was used to estimate verbal intelligence [37]. In this task, the child was asked to give definitions of words such as “umbrella” or “shoe.” The total score can be transformed to IQ scores, with 100 as the mean (SD 15).

### Statistics

Data were analyzed with IBM SPSS Statistics version 21.0.0.0. All outliers (scores with *z*>3.29) within the concerned group (ie, normally developing children and the ADHD group) were replaced by the mean + 3 times its standard deviation as advised by Field [38]. Means and standard deviations of all variables were calculated.

Potential age-related differences on Timo’s Adventure within the sample of normally developing children were examined by conducting Pearson correlation (for scale outcome measurements; eg, number of irrelevant mouse clicks, time used to complete the task) and Spearman correlation analyses (for the ordinal outcome measurements; eg, correct or incorrect, did the child choose the large or small reward). Age was used as a continuous variable in these analyses.

Second, a discriminant analysis was performed to investigate to which level the variables of the game can discriminate between children belonging to the ADHD group and the normally developing children. Sensitivity and specificity were measured. All children aged <6 years in the sample of normally developing children were excluded from these analyses, in order to match with the age of the children in the ADHD group, resulting in a control group of 57 children. An independent samples *t* test revealed that children in the ADHD group and children in the normally developing group were equal in terms of Vocabulary scores (ADHD group: mean 87.92, SD 13.05; control group: mean 94.74, SD 10.79; *t*_67_=1.92, *P*=.11), therefore it was not necessary to correct for IQ in further analyses.

Finally, it was analyzed on which variables children with ADHD scored significantly different scores from normally developing children. Again, all children aged <6 years in the sample of normally developing children were excluded from these analyses, in order to match with the age of the children in the ADHD group. Three types of analyses were used. For the continuous variables, general linear model univariate analyses were used. In four variables, the assumption of homogeneity in variances was violated; therefore, nonparametric *t* tests (Mann-Whitney) were used in these variables. In the categorical variables, Pearson chi-square analyses were performed.

## Results

### Descriptive Statistics of All Variables

Means and standard deviations for all variables of both groups are reported together with the analyses of group differences. All reported results are after correction for outliers.

### Developmental Effects

An overview of all correlation analyses between age and variables of Timo’s Adventure can be found in Table 2.

#### Pathway 1: Executive Functions

Pearson correlation analyses with age as a continuous variable showed significant correlations on 2 tasks: in the inhibition task (Monkey), older children had significantly fewer inhibition failures (*r*=−.33, *P*=.001); and in the reaction time task (Magic Land), older children collected significantly more stars (*r*=.60, *P*<.001) and were faster in collecting these stars (*r*=−.49, *P*<.001).

#### Pathway 2: Reward Mechanisms

No significant correlations were found in this pathway, indicating that age does not influence reward mechanisms.

#### Pathway 3: Time Perception

In the time production task (Balloon), older children produced significantly more correct balloons (ρ= .35, *P*<.001) and had more precise time productions than younger children (*r*=−.25, *P*=.01).

**Table 2:** Correlation between results on Timo’s Adventure and age for normally developing children (N=96).

Variables		Age^a^	*P* value
**Pathway 1: executive functions**			
	Dressing up, total score	ρ=−.08	.46
	Dressing up, clicks	*r*=−.05	.66
	Sandwich, total score	ρ=.21	.05
	Sandwich, clicks	*r*=.09	.71
	Balloon, clicks	*r*=.14	.18
	Monkey, failures	*r*=−.33	.001
	Monkey, clicks	*r*=.08	.45
	Magic Land, number of collected stars	*r*=.60	<.001
	Magic Land, average time for collected stars	*r*=−.49	<.001
	Magic Land, clicks	*r*=.16	.14
**Pathway 2: reward mechanisms**			
	Rocket, reward	ρ=.05	.65
	Rocket, time waited	*r*=.07	.50
**Pathway 3: time perception**			
	Balloon, correct	ρ=.35	<.001
	Balloon, average time for attempts	*r*=−.25	.01

^a^*r*: Pearson correlation; ρ: Spearman correlation.

**Table 3:** Structure matrix in discriminant analysis.

Output variable	Pooled within-group correlations between discriminating variables and standardized canonical discriminant functions
Magic Land, number of clicks	−.82
Magic Land, number of collected stars	.40
Balloon, number of clicks	.34
Monkey, number of failures	.31
Dressing up, total score	.29
Rocket, time waited	−.27
Balloon, number of correct balloons	−.18
Sandwich, total score	.17
Sandwich, number of clicks	.14
Magic Land, average reaction time for collected stars	.11
Rocket, small (=0) or large (=1) reward	−.10
Monkey, number of clicks	.09
Balloon, average time taken to inflate balloons	−.02
Dressing up, number of clicks	.01

### Differences Between Children With ADHD and Healthy Controls

#### Discriminant Analysis

All variables were included in a discriminant analysis to investigate whether Timo’s Adventure can discriminate between the children with ADHD and the healthy controls. A significant difference between the groups was found: Wilks Λ=.51, χ^2^_14_=50.8, *P*<.001. The structure matrix (see Table 3) revealed that especially the number of mouse clicks in several tasks and the mini-games on reaction time (Magic Land), inhibition (Monkey), and planning (Dressing up) were potential predictors. The classification results showed that 72% (41/57) children in the control group were correctly classified, and 88% (35/40) of the children in the ADHD group were correctly classified as having ADHD. Overall, 78% (76/97) of the children were correctly classified as being in the ADHD group or in the control group. Sensitivity of Timo’s Adventure was 0.89 and specificity was 0.69.

#### Group Differences on Individual Variables

Because the combination of all variables was useful in discriminating between children with ADHD and normally developing children, the specific variables for which children with ADHD had a different result from normally developing children were examined. All significant differences between children with ADHD and healthy controls are reported.

##### Pathway 1: Executive Functions

Results of this pathway can be found in Table 4. There was a significant association between the group (ADHD or control) and the score on the Dressing up task (planning; χ^2^_2_=11.4, *P*=.003, *V*=.35), indicating that typically developing children had better scores than children with ADHD on a planning task. In the Magic Land task (simple reaction time), children with ADHD used more mouse clicks in collecting stars than children in the control group (*U*=389.50, *P*<.001, *r*=−.55).

**Table 4:** Results on Timo’s Adventure for normally developing children in the control group (N=56) and children in the attention-deficit/hyperactivity disorder group (N=40) in pathway 1, executive functions.

Variable	Group	Mean	SD	Statistic	*P* value	Effect size
**Dressing up, total score (0-2)**						
	Controls	1.18	0.56	χ^2^_2_=11.41	.003	*V*^a^=.35
	ADHD^b^	0.93	0.81			
**Dressing up, number of clicks (minimum for a satisfying result is 4 clicks)**						
	Controls	17.59	14.32	*U*^c^=895.00	.09	*r*^d^=−.17
	ADHD	26.14	22.61			
**Sandwich, total score (0-5)**						
	Controls	0.89	1.10	*F*^f^_1,95_=0.47	.50	Ƞ_p_^2^=.01^e^
	ADHD	1.05	0.97			
**Sandwich, number of clicks (minimum for 5 correct sandwiches is 22 clicks)**						
	Controls	87.49	51.12	*U*=823.00	.25	*r*=−.12
	ADHD	92.57	53.48			
**Balloon, number of clicks (minimum for 3 correct balloons is 6)**						
	Controls	108.52	93.91	*U*=945.50	.24	*r*=−.12
	ADHD	206.87	227.87			
**Monkey, number of failures (minimum is 0)**						
	Controls	0.77	1.33	*F*_1,96_=2.97	.09	Ƞ_p_^2^=.03
	ADHD	1.26	1.48			
**Monkey, number of clicks (minimum to complete the task is 6)**						
	Controls	29.72	2.20	*F*_1,96_=0.82	.37	Ƞ_p_^2^=.01
	ADHD	33.83	1.62			
**Magic Land, number of collected stars (maximum is 50)**						
	Controls	37.61	10.87	*F*_1,95_=2.92	.09	Ƞ_p_^2^=0.03
	ADHD	41.41	8.33			
**Magic Land, average reaction time for collected stars**						
	Controls	2.25	0.55	*F*_1,95_=0.02	.88	Ƞ_p_^2^=0.00
	ADHD	2.22	0.48			
**Magic Land, number of clicks**						
	Controls	76.27	19.52	*U*=389.50	<.001	*r*=−.55
	ADHD	155.65	89.68			

^a^*V*: Cramer’s *V*.

^b^ADHD: attention-deficit/hyperactivity disorder.

^c^*U*: Mann-Whitney test.

^d^*r*: Pearson correlation coefficient.

^e^Ƞ_p_^2^: partial variance explained.

**Table 5:** Results on Timo’s Adventure for normally developing children in the control group (N=56) and children in the attention-deficit/hyperactivity disorder group (N=40) in pathway 2, reward mechanisms.

Variable	Group	Mean	SD	Statistic	*P* value	Effect size
**Rocket, small (=0) or large (=1) reward**						
	Controls	0.55	0.50	χ^2^_1_=7.3	.01	*V*^a^=.28
	ADHD^b^	0.28	0.46			
**Rocket, time waited (minimum is 0 seconds, maximum is 120 seconds)**						
	Controls	80.98	50.05	*F*_1,92_=5.52	.02	Ƞ_p_^2^=.06^d^
	ADHD	56.26	50.13			

^a^*V*: Cramer’s *V*.

^b^ADHD: attention-deficit/hyperactivity disorder.

^c^Ƞ_p_^2^: partial variance explained.

##### Pathway 2: Reward Mechanisms

Results of this pathway can be found in Table 5. In the Rocket task, a significant association between the group (ADHD or control) and whether or not a child chose the delayed reward was found (χ^2^_1_=7.3, *P*=.01, *V*=.28). Also, the total time that the child waited before he or she ended the task was significantly different: children in the control group were able to wait longer for the delayed reward than the children in the ADHD group (*F*_1,92_ =5.52, *P*=.02, Ƞ_p_^2^=.06).

##### Pathway 3: Time Perception

Results of this pathway can be found in Table 6. No significant differences between children with ADHD and normally developing children were found in the time production task.

**Table 6:** Results on Timo’s Adventure for normally developing children in the control group (N=56) and children in the attention-deficit/hyperactivity disorder group (N=40) in pathway 3, time perception.

Variable	Group	Mean	SD	Statistic	*P* value	Effect size
**Balloon, number of correct balloons (minimum is 0, maximum is 3)**						
	Controls	2.25	1.09	*F*_1,96_=0.07	.80	Ƞ_p_^2^=.00^a^
	ADHD^b^	2.21	1.06			
**Balloon, average time taken to inflate balloons**						
	Controls	4.33	4.05	*F*_1,96_=1.69	.20	Ƞ_p_^2^=.02
	ADHD	3.38	2.01			

^b^Ƞ_p_^2^: partial variance explained.

^c^ADHD: attention-deficit/hyperactivity disorder.

## Discussion

### Principal Findings

Recently, the development and use of computerized tasks in measuring, for example, neurocognitive abilities is increasing and results in promising effects in the field of interventions. For instance, children with ADHD benefit from game-based training tools on executive functions such as Braingame Brian and Cogmed, as was reported in a review by Peijnenborgh et al [39]. Important elements of these training tools are believed to be the use of fantasy, a story line, adaptation of the degree of difficulty, and the use of immediate rewards. However, most studies focus on training tools, whereas in our research a diagnostic tool was studied. This computerized diagnostic tool, named Timo’s Adventure, was developed for young children (between 4 and 8 years old) to investigate the presence of the 3 distinct patterns of possible deficits in ADHD as described in the triple-pathway model by Sonuga-Barke and colleagues. To our knowledge, this is the first computerized tool in which all 3 pathways are assessed. The aim of this study was to investigate the clinical validity of Timo’s Adventure.

The first proof of validity was found in the developmental effects of the game. In a population of 96 normally developing children between 4 and 8 years old, we found significant correlations with age in 2 of the 3 pathways. The older the child, the faster he or she is in completing the tasks. Furthermore, it was found that older children are better in inhibiting their response on a go or no-go task. This is in line with previous research (eg, [40,41]). Also, in a reaction time task, older children have better responses on alertness and have better reactions to visually presented stimuli after a visual warning signal. Again, this is in line with previous research (eg, [42]). Furthermore, age-related differences were found in the third pathway (ie, time perception), indicating that the older the child, the better he or she is in producing a predetermined time interval. This is in line with research by, for example, Friedman and Laycock [43] and Pouthas and Jacquet [13] stating that development of time perception skills increases sharply at an early age (ie, before the age of 7 years) and refines in the last grades of elementary school. Interestingly, no developmental effects were found in the second pathway (reward mechanisms), indicating that age does not influence the choice between immediate or delayed rewards. This might be caused by the fact that this aspect might be fully developed before the age of 4 years, as the findings by Mischel et al [44] suggest.

Further proof for clinical validity was found in satisfying results on the discriminant analysis, indicating that Timo’s Adventure was correct in most classifications. Sensitivity and specificity of the measurement were satisfying. Our results are similar to, and sometimes even more promising than, other diagnostic measurements. For instance, Williams and colleagues [45] could classify 68% of the children with ADHD correctly when using IntegNeuro. However, only 2 of the 3 pathways are included in IntegNeuro, and it is not suitable for young children.

When looking more closely at the individual variables that help to differentiate between children with and without ADHD, we found several significant differences between both groups. In the first pathway (executive functions), we found that children with ADHD have significantly more irrelevant mouse clicks on the Magic Land task (reaction time) than healthy peers. This indicates impulsiveness, motor restlessness, and hyperactive behavior, as is also suggested by Hervey and colleagues [46]. Also, children with ADHD had lower planning skills than healthy controls had, what might be expected because planning and organization are affected in children with ADHD [47]. In several tasks we found results that were encouraging but not statistically significant (eg, children with ADHD tend to have more inhibition mistakes in the Monkey task and collect more stars in the Magic Land than typically developing children). This seems promising for the future: maybe, with some adjustments to the tasks, sensitivity and specificity can even increase.

Evidence was also found for differences between the ADHD group and the controls in the second pathway (reward mechanisms): children with ADHD chose the large but delayed reward less often than the control group and did not wait as long as the control group before deciding to end the task. This is in line with previous research, which states that children with ADHD have an aversion for delay [48]. No significant differences were found in the third pathway of Sonuga-Barke’s model (time perception). Although timing deficits are known in children with ADHD, it is not uncommon that time production tasks do not result in significant effects [49]. Further research is necessary to gain more information on this aspect of possible ADHD-related deficits.

Finally, we found that user experiences were positive: when asked afterward, 81% of the children said they liked the game very much, and an extra 14% of the children said that they liked the game.

One limitation of this study is that information on reliability cannot be reported at this moment. Because Timo’s Adventure consists of several independent functions, analysis of Cronbach alpha would automatically result in low consistency between the items. It would be interesting to test children several times, to collect data for test-retest reliability analyses. Further research is necessary to examine the reliability of this instrument. Another interesting question for future research might be to investigate possible effects of use of medication. In our analysis all children with ADHD who were taking medication (18 in total) were excluded, but it might be possible that medication influences only one (or a combination) of the pathways. Finally, it would be interesting to determine which (or combination of) tasks and corresponding outcome measurements are especially sensitive for the diagnosis of ADHD. It is possible that a total score and normative data can be measured, which can be used to determine a profile of ADHD symptoms. Future research is necessary to determine such a profile or total score.

### Conclusions

This is the first time that all 3 pathways of Sonuga-Barke’s model are included in one diagnostic computerized tool with a context of rewards and story line. In clinical care, diagnostic instruments on time perception and reward mechanisms are scarce, but it is necessary to gain information on these aspects to complete an analysis of strengths and weaknesses of the child. Proof for validity of Timo’s Adventure was found in developmental effects and group differences between normally developing children and children with ADHD, and Timo’s Adventure was satisfying accurately when classifying to which group (ie, the ADHD group or the healthy controls) the child belonged. This suggests that Timo’s Adventure can be of added value in the diagnosis of ADHD because it helps in formulating a profile of strengths and weaknesses. Further research is necessary to confirm these findings and to examine potential effects of medication.

## Abbreviations

ADHD: attention-deficit/hyperactivity disorder
DSM-V: Diagnostic and Statistical Manual of Mental Disorders (Fifth Edition)
WPPSI-III-NL: Wechsler Preschool and Primary Scale of Intelligence, Dutch version
